# Impact of AI on radiology: a EuroAIM/EuSoMII 2024 survey among members of the European Society of Radiology

**DOI:** 10.1186/s13244-024-01801-w

**Published:** 2024-10-07

**Authors:** Moreno Zanardo, Jacob J. Visser, Anna Colarieti, Renato Cuocolo, Michail E. Klontzas, Daniel Pinto dos Santos, Francesco Sardanelli, European Society of Radiology (ESR)

**Affiliations:** 1 Unit of Radiology, IRCCS Policlinico San Donato, San Donato Milanese, Italy; 2 Department of Radiology & Nuclear Medicine, Erasmus MC, Rotterdam, The Netherlands; 3 Department of Medicine, Surgery, and Dentistry, University of Salerno, Baronissi, Italy; 4 Department of Radiology, School of Medicine, University of Crete, Heraklion, Greece; 5 Division of Radiology, Department of Clinical Science, Intervention and Technology (CLINTEC), Karolinska Institutet, Stockholm, Sweden; 6 Computational Biomedicine Laboratory, Institute of Computer Science, Foundation for Research and Technology (ICS-FORTH), Crete, Greece; 7 Department of Radiology, University Hospital of Frankfurt, Frankfurt, Germany; 8 Department of Radiology, University Hospital of Cologne, Cologne, Germany; 9 Lega Italiana per la Lotta contro i Tumori (LILT) Milano Monza Brianza, Milan, Italy; 10 Am Gestade 1, Vienna, Austria

**Keywords:** Artificial intelligence, Radiology, Diagnostic imaging, Surveys and questionnaires

## Abstract

**Abstract:**

In order to assess the perceptions and expectations of the radiology staff about artificial intelligence (AI), we conducted an online survey among ESR members (January–March 2024). It was designed considering that conducted in 2018, updated according to recent advancements and emerging topics, consisting of seven questions regarding demographics and professional background and 28 AI questions. Of 28,000 members contacted, 572 (2%) completed the survey. AI impact was predominantly expected on breast and oncologic imaging, primarily involving CT, mammography, and MRI, and in the detection of abnormalities in asymptomatic subjects. About half of responders did not foresee an impact of AI on job opportunities. For 273/572 respondents (48%), AI-only reports would not be accepted by patients; and 242/572 respondents (42%) think that the use of AI systems will not change the relationship between the radiological team and the patient. According to 255/572 respondents (45%), radiologists will take responsibility for any AI output that may influence clinical decision-making. Of 572 respondents, 274 (48%) are currently using AI, 153 (27%) are not, and 145 (25%) are planning to do so. In conclusion, ESR members declare familiarity with AI technologies, as well as recognition of their potential benefits and challenges. Compared to the 2018 survey, the perception of AI's impact on job opportunities is in general slightly less optimistic (more positive from AI users/researchers), while the radiologist’s responsibility for AI outputs is confirmed. The use of large language models is declared not only limited to research, highlighting the need for education in AI and its regulations.

**Critical relevance statement:**

This study critically evaluates the current impact of AI on radiology, revealing significant usage patterns and clinical implications, thereby guiding future integration strategies to enhance efficiency and patient care in clinical radiology.

**Key Points:**

- The survey examines ESR member's views about the impact of AI on radiology practice.
- AI use is relevant in CT and MRI, with varying impacts on job roles.
- AI tools enhance clinical efficiency but require radiologist oversight for patient acceptance.

**Graphical Abstract:**

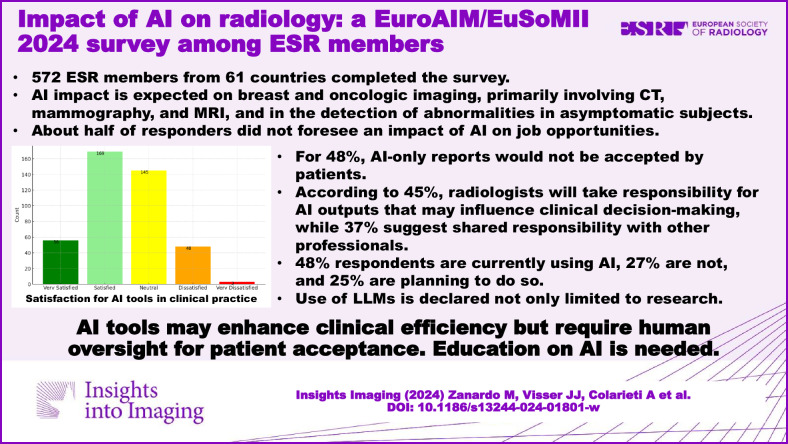

## Background

In 2019, the European Society of Radiology (ESR)/European Network for the Assessment of Imaging in Medicine (EuroAIM) published the first survey on the impact of artificial intelligence (AI) on radiology [1]. Since then, AI has gained remarkable interest for its applications to radiology. AI systems, based on machine learning or deep learning are showing potential for image acquisition, pre- and post-processing, and, ultimately, disease risk stratification and clinical decision-making [2–4], as also happened with the recent COVID-19 pandemic [5]. A notable increase in AI usage was also reported between 2018 and 2022 among members of the ESR [6].

The debate about AI’s impact on biomedicine and healthcare has grown, with an obvious focus on radiology. In fact, in addition to the interest in radiomics (“images are more than pictures, they are data” [7]), machine/deep learning was early applied to medical images from reconstruction to clinical prediction and diagnosis [8, 9]. Such a transformative innovation raised hopes and fears within the radiological community and beyond [6, 10]. In the last couple of years, a substantial step forward has been the advent of generative AI, with large language models (LLMs) and foundation models beyond language, enabling the automatic creation of images and videos, as well as image interpretation [11].

When applied to radiology, AI tools are considered equivalent to medical devices, with related ethical and regulatory issues [12], as recently defined also by the AI Act from the European Union Parliament (https://artificialintelligenceact.eu). Many efforts were dedicated to the standardization of reporting of AI studies, with guidelines and checklists available [13–17]. Of note is that the editors of the ESR journals published a statement about the use of LLMs by authors, reviewers, and editors [18].

Radiologists’ views and perceptions about AI applications to medical imaging have been reported [3, 19–24]. Our aim was to capture the current opinions and experiences of ESR members regarding the impact of AI on radiology with a new online survey. This study seeks to provide insights into how perceptions and uses of AI have changed over the past six years. By comparing the findings from 2018 [1] and 2024, we aim to understand the evolving impact of AI on radiology practices, job roles, and the professional landscape within the ESR community. This longitudinal analysis is crucial for identifying trends, assessing the progress of AI integration, and informing future strategies for AI implementation and educational needs to be addressed by the ESR.

## Methods

### Survey design

The survey design was defined taking into account that previously conducted in 2018 [1], updated to reflect recent advancements and emerging topics. To facilitate a meaningful comparison with the 2018 survey, we retained 15 core questions that addressed fundamental aspects of AI’s impact on radiology. Thirteen new questions were introduced to address recent advancements in AI technology, including the rise of generative AI, LLMs, and updated regulatory frameworks. The new questions were proposed by the EuroAIM working group and reviewed and integrated by the European Society of Medical Imaging Informatics (EuSoMII) working group, as well as, by the dedicated group of the ESR. The survey consisted of three parts: seven questions about demographics and professional background (I–VII); and 28 multiple-choice questions on AI issues; of them, 15 were identical to those posed in the 2018 survey (1–15) and 13 were new questions (16–28). The survey mandated that all questions be answered before submission. The full questionnaire is reported in the Supplementary material.

### Survey promotion and administration

The survey was promoted by the EuroAIM, the EuSoMII, and the EFRS - European Federation of Radiographer Societies, under the umbrella of the ESR. Participation was voluntary, and no personal identifying information was collected, ensuring respondent anonymity. The survey was conducted in accordance with the ESR ethical standards and adhered to GDPR regulations for data protection and privacy. It was administered between January and March 2024, and intended to be completed in about 10 min. Invitation to participate was emailed to ESR members, reaching approximately 28,000 individuals. The e-mail contained a URL link to the survey, hosted on the Google form platform. Two reminder emails were sent on January 26 and March 18, 2024. The survey was closed on March 31, 2024.

### Data collection and analysis

Survey responses were automatically recorded and processed using Excel® (Microsoft, RedMond, WA, USA). Descriptive statistics, including frequencies and percentages, were calculated for all categorical variables. Association analysis was performed using the χ^2^ test. For the comparison of answers on AI given in 2024 to those in 2018, 95% confidence intervals (CI) were calculated using Excel® (Microsoft, RedMond, WA, USA).

## Results

### Demographics and professional profiles (questions from I to VII)

**Fig. 1:**
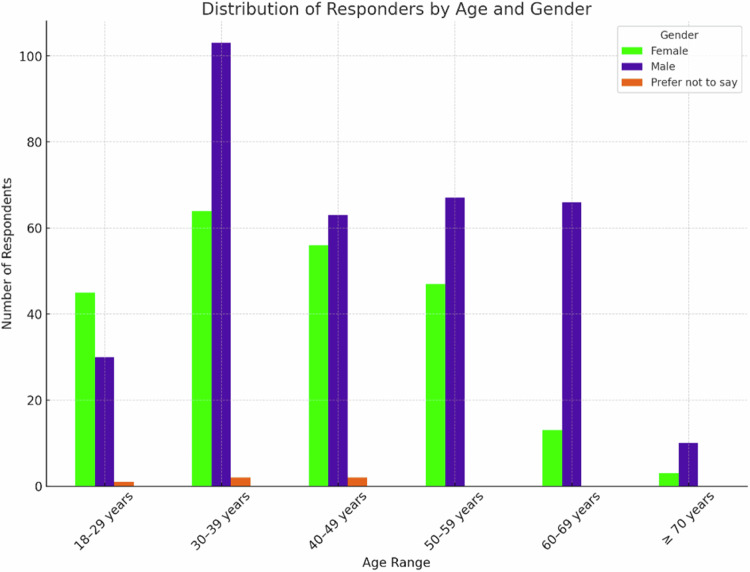
Distribution of responders by age and gender (“Prefer not to say” answers, *n* = 5, 0.8%)

A total of 572 responders out of 28,000 sent e-mails completed the whole survey (2.0%). Among 572 survey responders, 350 (61.2%) were radiologists, 87 (15.2%) were radiology residents, 84 (14.7%) were radiographers, 19 (3.3%) physicists, 32 (5.6%) other categories (mainly physicians with a non-radiological specialization and medical students). The working places were: university/teaching hospital for 314 (54.9%) responders; hospital for 189 (33.0%); private practice for 37 (6.5%); private company for 20 (3.5%); and private research center for 7 (1.2%). Information about gender distribution according to age classes is reported in Fig. 1. Of 572 respondents, males were 339 (59.3%), females 228 (39.9%), while 5 (0.8%) answered: “Prefer not to say”.

Responses came from 61 countries with the following distribution per continent: Europe: 535 (93.5%); Asia: 29 (5.1%); Africa: 3 (0.5%); North America: 3 (0.5%); South America: 1 (0.2%); and Australia: 1 (0.2%). Data on country distribution is detailed in the Supplementary File.

### AI and medical imaging (questions from 1 to 3)

**Fig. 2:**
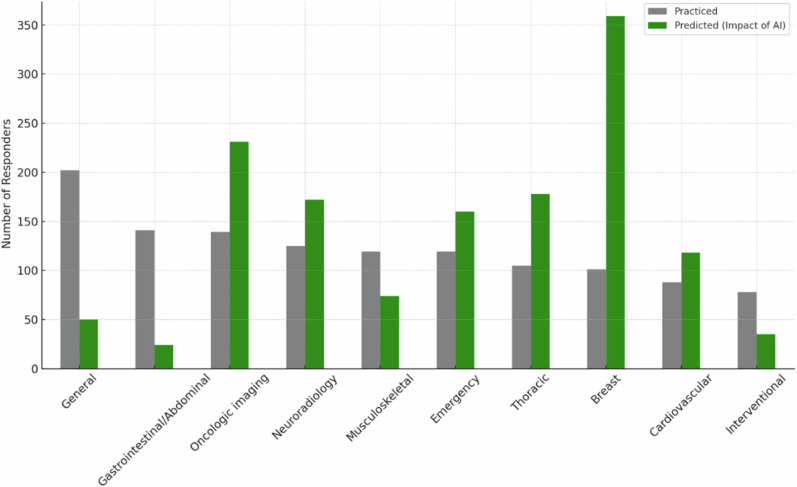
Distribution of responders according to subspecialties, practised and predicted to be impacted by AI. The gray bars represent the number of responders that practice each subspecialty (sorted in decreasing order), while the green bars represent those who foresaw an impact of AI on each subspecialty

**Fig. 3:**
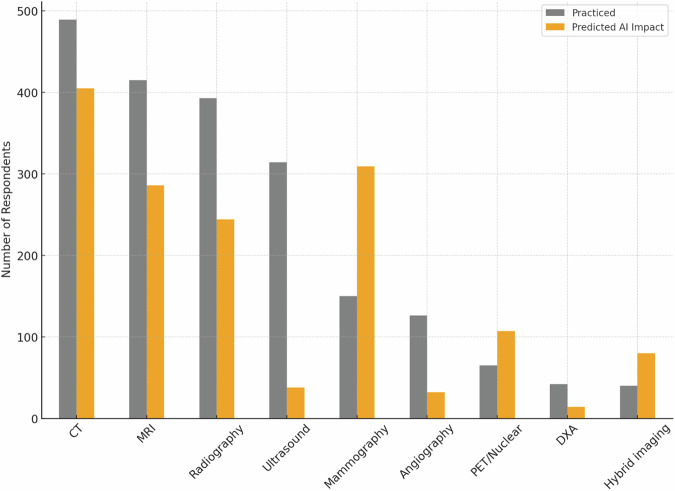
Distribution of responders according to imaging modalities, practised and predicted to be impacted by AI. Gray bars represent the number of responders practising each modality (sorted in decreasing order), while the orange bars represent those who believe that that modality will be impacted by AI applications. CT, computed tomography; MRI, magnetic resonance imaging; PET, positron emission tomography; DXA, dual-energy X-ray absorptiometry

The distribution of responders according to radiology subspecialty and their opinion about which subspecialties will be mostly influenced by the introduction of AI systems is reported in Fig. 2. Responders’ distribution according to practised imaging modalities and their opinion about which imaging modality will be used most to provide input data for AI systems is shown in Fig. 3. Table 1 shows AI application judged by the responders as most relevant in radiology and their corresponding rates by responders.

**Table 1:** Relevant applications of AI in radiology by 572 responders

AI application	Respondents	
	Number	Percentage
Detection in asymptomatic subjects (screening)	298	52.1%
Image postprocessing	271	47.4%
Imaging protocols optimization	227	39.7%
Staging/restaging in oncology	208	36.4%
Support for structured reporting	161	28.1%
Quantitative measure of imaging biomarkers	157	27.5%
Detection of incidental findings	150	26.2%
Lesion characterization/diagnosis in symptomatic subjects	134	23.4%
Prognosis	31	5.4%

The sum of the percentages is superior to 100% due to the possibility of responders selecting up to three options

### AI impact on radiological work (questions from 4 to 10)

About half of 572 responders do not foresee an impact of AI in terms of job opportunities (295, 51.6%), while 162 (28.3%) expect a reduction in job opportunities, and the remaining 115 (20.1%) expect an increase. Tables 2 and 3 show the subgroup analyses according to being/not being AI users or researchers.

**Table 2:** Association between expected variations in job positions due to AI with being/not being AI users

	Expected job positions increase	Excepted job positions unchanged	Expected job position reduction	Total	*p*-value*
Users of AI-based products or services in clinical practice	65 (23.7%)	140 (51.1%)	69 (25.2%)	274 (100%)	0.071
Nonusers of AI-based products or services in clinical practice	50 (16.8%)	155 (52.0%)	93 (31.2%)	298 (100%)	
Total	115 (20.1%)	295 (51.6%)	162 (28.3%)	572 (100%)	

^* ^χ^2^ test

**Table 3:** Association between expected variations of job positions due to AI with being/not being AI researchers

	Expected job positions increase	Excepted job positions unchanged	Expected job position reduction	Total	*p*-value*
Researchers on AI-based products or services in clinical practice	49 (22.8%)	114 (53.0%)	52 (24.2%)	215 (100%)	0.178
Not researchers on AI-based products or services in clinical practice	66 (18.5%)	181 (50.7%)	110 (30.8%)	357 (100%)	
Total	115 (20.1%)	295 (51.6%)	162 (28.3%)	572 (100%)	

^* ^χ^2^ test

The use of AI-based applications will make the radiological team’s duties more clinical according to 256 (44.8%) responders, more technical for 208 (36.4%), unchanged for 92 (16.1%), and both more technical and clinical for 16 (2.8%). For the relative majority of the responders (229, 40.0%), radiologists will be more focused on radiology subspecialties and therefore AI-based applications will not help to report examinations outside the field of subspecialization or will not change the current practice (207, 36.2%), while 136 (23.8%) think that radiologists will be less focused on radiology subspecialties. Regarding reporting workload, 270 (47.2%) expect an impact in terms of increased total reporting workload, while 184 (32.2%) expect a reduced reporting workload, and 118 (20.6%) expect no impact.

The legal responsibility of AI systems outcome will be taken by radiologists alone for 255 (44.6%) responders, will be “shared” for 211 (36.8%), and will be taken by AI developers for 68 (11.9%), insurance companies for 21 (3.7%), other physicians (e.g. clinicians asking for the exam) for 13 (2.3%) (four other answers, 0.7%). Of the 572 responders, 273 (47.7%) believe that patients will not accept a report made by an AI application alone without supervision and approval by a physician, while 92 (16.1%) believe that an AI alone report will be accepted; on the other hand, 207 (36.2%) believe it is now too early to estimate patients’ reaction to this scenario.

Among responders, 242 (42.3%) think that the use of AI systems will not change the relationship between the radiological team and the patient, while 191 (33.4%) believe it will become more interactive and 139 (24.3%) expect that it will become more impersonal.

### Radiological team involvement in AI tools development (questions from 11 to 15)

Among 572 responders, 559 (97.7%) believe that the radiological team will play a role in the development and validation of AI applications to medical imaging, in particular, the relative majority believes that they should supervise all development stages of an AI system applied to radiology (355, 62.1%). Specific tasks were rated as follows: helping in task definition (256, 44.8%), providing labelled images (181, 31.6%), and developing AI-based applications (133, 23.3%).

The relative majority of the responders would like to be educated on advantages and limitations of AI applications (410, 71.7%), on the clinical use of AI applications (357, 62.4%), on technical methods (142, 24.8%), on how to get into the driver’s seat in using AI (134, 23.4%), on how to survive to the AI revolution (48, 8.4%), and on how to avoid the use of AI applications (14, 2.4%).

Moreover, responders believe that if AI systems will allow to save working/reporting time, they should be used to interact with: other clinicians (228, 39.9%), patients (206, 36.0%), AI developers (e.g. engineers, computer scientists) (83, 14.5%), other radiologists (53, 9.3%), or administrators (2, 0.3%).

Of 572 responders, 274 (47.9%) are currently using AI systems in their clinical practice, while 153 (26.8%) do not use them, and 145 (25.3%) do not use them at present but are planning to do it. Meanwhile, 239 of 572 responders (41.8%) are currently not involved in research projects on AI applications development, 118 (20.6%) are planning to be involved, 113 (19.8%) are currently involved in AI systems development, and 102 (17.8%) in their testing.

### Usage and perception of AI tools (questions from 16 to 23)

**Fig. 4:**
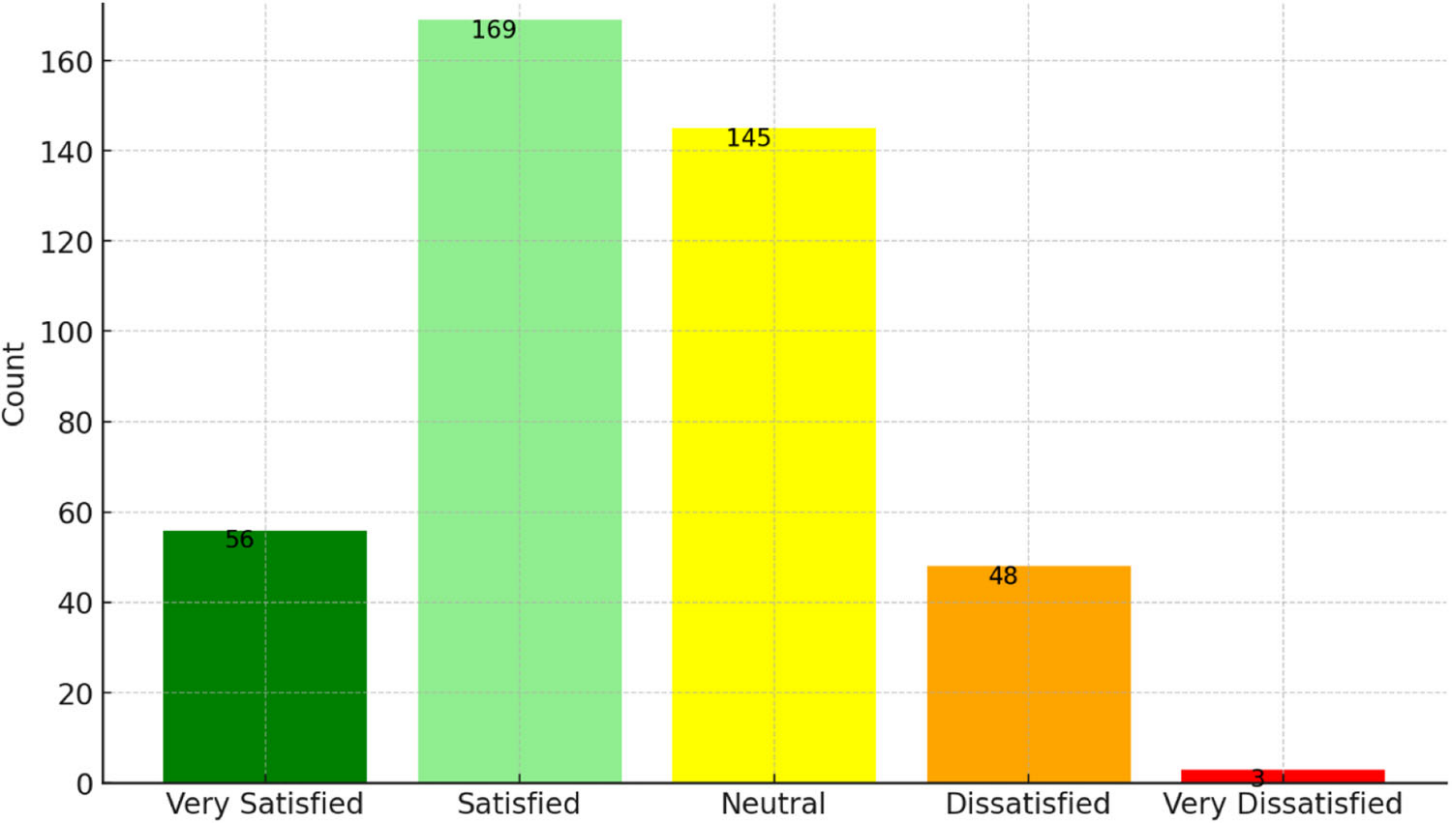
Bar chart representing the distribution of satisfaction levels regarding the performance of certified AI tools used by responders in clinical practice

Radiological team members that never used any AI tools were 151/572 (26.4%); 56/421 (13.3%) were very satisfied, 169/421 (40.1%) were satisfied, 145/421 (34.5%) were neutral, 48/421 (11.4%) dissatisfied and 3/421 (0.7%) very dissatisfied (Fig. 4).

The most common radiological modalities for certified AI tools used in clinical practice are CT (222, 38.8%) and radiography (137, 24.0%), with MRI (134, 23.4%) and mammography (75, 13.1%) also being frequently mentioned.

The survey results indicate that 169/572 (29.6%) of respondents are not familiar with the classification under the medical devices regulation and postmarket surveillance requirements for the certified AI tools they are using. Meanwhile, 110/572 (19.2%) are familiar with these requirements, while an equal 110/572 (19.2%) do not know the classification and 31/572 (5.4%) do not know the postmarket surveillance requirements;152/572 (26.6%) declared no current or previous use of these tools.^1^ Data on the association between being familiar/not familiar with medical devices regulation and postmarket surveillance requirements for certified AI tools among responders being/not being AI users are reported in Table 4.

**Table 4:** Association between being familiar/not familiar with medical devices regulation and postmarket surveillance requirements for certified AI tools among responders being/not being AI users

	Being familiar	Being not familiar	Total	*p*-value*
Users of AI-based products or services in clinical practice	79 (28.8%)	195 (71.2%)	274 (100%)	< 0.001
Nonusers of AI-based products or services in clinical practice	31 (10.4%)	267 (89.6%)	298 (100%)	
Total	110 (19.2%)	462 (80.8%)	572 (100%)	

^* ^χ^2^ test

The survey results indicate that the most common sources for staying informed about AI are conferences and congresses (428, 74.8%), followed by scientific papers (382, 66.8%), and colleagues (242, 42.3%). Social media (206, 36.0%) and newsletters (197, 34.4%) are also significant sources, while books (69, 12.1%) and AI itself (64, 11.2%) are less common. Other sources like vendors, podcasts, and industry-related information are mentioned less frequently.

Among 572 respondents, 354 (61.9%) declare that their department does not have a dedicated budget for AI, while 164 (28.7%) of respondents are unsure. Only 54 (9.4%) of respondents indicated that their department has a dedicated budget for AI, mainly based in Italy, Germany, Netherlands and Switzerland.

The survey results indicate that 286/572 respondents (50%) are not involved in any AI research. The tasks/roles declared by those involved in AI research are reported in Table 5.

**Table 5:** Tasks/roles declared by 286 responders involved in AI research

Tasks/roles	Respondents	
	Number	Percentage
Image annotation/segmentation (and or provider of annotated datasets)	135	47.2%
Cooperation in writing of scientific/clinical papers	127	44.4%
Supervision training and internal validation	126	44.1%
Proponent (identifier of clinical needs)	113	39.5%
Developer (direct interaction and collaboration with data scientists and/or IT specialists to develop AI models)	104	36.4%
Collaboration in external validation	104	36.4%
Data selector and provider	101	35.3%

The sum of the percentages is superior to 100% due to the possibility of responders selecting up to three options

*AI* artificial intelligence, *IT* information technology

The relative majority of respondents (341/572, 59.6%) believe that the Covid-19 pandemic accelerated the development or implementation of AI research in medical imaging, while 129/572 (22.6%) are unsure, and 102/572 (17.8%) do not believe it had an accelerating effect.

The main potential barriers to AI implementation in clinical practice are costs/lack of budget opted by 283 (49.5%) responders, legal issues (250, 43.7%), lack of validation/scientific evidence (203, 35.5%), lack of vision/policy/ownership (157, 27.4%), and information technology and systems integration (137, 24.0%). Other barriers such as low efficacy, lack of reimbursement, and various less common concerns were mentioned by less than 2% of respondents each.

### LLM perception and utilization (questions from 24 to 28)

The survey results indicate that 299/572 respondents (52.3%) do not currently use LLMs in their practice but are interested in doing so. Meanwhile, 124/572 (21.7%) use them occasionally, 122/572 (21.3%) are not interested, and 27/572 (4.7%) use them often. Among those who answered (151, 26.4%) positively, the most common settings for using LLMs in clinical practice are: research activities/scientific writing (98/151, 64.9%), literature review (80/151, 53.0%), risk assessment (19/151, 12.6%), image annotation and reporting (18/151, 11.9%), and screening/diagnosis (17/151, 11.2%). The main potential benefits of using LLMs in radiology are access to up-to-date medical literature and research (241/572, 42.1%), enhanced efficiency in image interpretation (183/572, 32.0%), improved diagnostic accuracy (101/572, 17.7%), cost savings (80/572, 14.0%), and enhanced efficiency in imaging procedure execution (78/572, 13.6%). Additionally, 199/572 (34.8%) respondents are unsure or have no opinion on the potential LLMs benefits.

The main concerns or reservations about incorporating LLMs into clinical practice are AI model reliability and bias (318/572, 55.6%), legal and ethical implications (266/572, 46.5%), and data privacy vulnerability (181/572, 31.6%). Additionally, 64/572 of respondents (11.2%) have no concerns or reservations, while 44/572 (7.7%) are worried about the negative impact on the radiological team.

Among the 572 respondents, 380 (66.4%) have not used AI imaging generative models (e.g. ChatGPT 4o, Midjourney, DALL-E, or Adobe Firefly) in their clinical practice or for academic purposes but are interested in doing so. Additionally, 119/572 (20.8%) have not used these models and are not interested, while 59/572 (10.3%) use them occasionally, and 14/572 (2.5%) use them often.

The comparisons between answers on AI given in the current survey with those given in 2018 judged to be relevant for outlining the current trends are provided in Table 6 with their 95% CI for the two surveys.

**Table 6:** Comparison of answers to the survey about AI in radiology: 2024 vs 2018

Variable/question		2018	2024	Δ
Rate of responders		675/24.000 2.8% (2.6–3.0%)	572/28.000 2.0% (1.9–2.2%)	−**0.8%**
Do you foresee an AI impact on a professional radiologist’s life in terms of the number of job positions in the next 5–10 years?	Yes, job positions will increase	218/675 32.3% (28.8–36.0%)	115/572 20.1% (16.9–23.6%)	−**12.2%**
	Yes, job positions will be reduced	157/675 23.3% (20.1–26.6%)	162/572 28.3% (24.7–32.2%)	+5.0%
	No	300/675 44.4% (40.7–48.3%)	295/572 51.6% (47.4–55.7%)	+7.2%
In the next 5–10 years, who will take the legal responsibility for AI-system output?	Radiologists	277/675 41.0% (37.3–44.9%)	255/572 44.6% (40.5–48.8%)	+3.6%
	Other physicians (e.g. clinicians requesting the imaging study)	7/675 1.0% (0.4–2.1%)	13/572 2.3% (1.2–3.9%)	+1.3%
	Developers of AI applications	69/675 10.2% (8.0–12.8%)	68/572 11.9% (9.4–14.8%)	+1.7%
	Insurance companies	24/675 3.6% (2.3–5.2%)	21/572 3.7% (2.3–5.6%)	+0.1%
	Shared responsibility	277/675 41.0% (37.3–44.9%)	211/572 36.9% (32.9–41.0%)	−4.1%
	Other	21/675 3.1% (1.9–4.7%)	4/572 0.7% (0.2–1.8%)	−**2.4%**
In the next 5–10 years, will patients mostly accept a report from AI applications without supervision and approval by a physician?	Yes	79/675 11.7% (9.4–14.4%)	92/572 16.1% (13.2–19.4%)	+4.4%
	No	374/675 55.4% (51.6–59.2%)	273/572 47.7% (43.6–51.9%)	−7.7%
	Difficult to estimate at present	222/675 32.9% (29.4–36.6%)	207/572 36.2% (32.2–40.3%)	+3.3%
What will be the role of radiologists in developing/validating AI applications for medical imaging?	None	0/675 0.0% (0.0–0.5%)	13/572 2.3% (1.2–3.9%)	**+2.3%**
	Provide labelled images	197/675 29% (25.8–32.8%)	181/572 31.6% 27.8–35.6%)	+2.6%
	Help in task definition	359/675 53.2% (49.3–57.0%)	256/572 44.8% (40.6–48.9%)	−**8.4%**
	Develop AI-based applications	188/675 27.9% (24.5–31.4%)	133/572 23.3% (19.8–26.9%)	−4.6%
	Supervise all stages needed to develop an AI-based application	434/675 64.3% (60.6–67.9%)	335/572 62.1% (57.9–66.1%)	−2.2%
Are you utilizing AI-based products or services in your clinical practice?	Yes	138/675 20.4% (17.5–23.7%)	274/572 47.9% (43.7–52.1%)	**+27.5%**
	No, but planning to utilise	205/675 30.4% (26.9–34.0%)	145/572 25.3% (21.8–29.1%)	−5.1%
	No	321/675 47.6% (43.7–51.4%)	153/572 26.8% (23.2–30.6%)	−**20.8%**
Should radiologists be educated on:	Technical methods (e.g. machine/deep learning algorithms)	119/675 17.6% (14.8–20.7%)	142/572 24.8% (21.3–28.6%)	**+7.2%**
	Advantages and limitations of AI applications	463/675 68.6% (64.9–72.1%)	410/572 71.7% (67.8–75.3%)	+3.1%
	Clinical use of AI applications	392/675 58.1% (54.2–61.8%)	357/572 62.4% (58.3–66.4%)	+4.3%
	How to get into the driver’s seat using AI	228/675 33.8% (30.2–37.5%)	134/572 23.4% (20.0–27.1%)	−**10.4%**
	How to avoid the use of AI applications	6/675 0.9% (0.3–1.9%)	14/572 2.4% (1.3–4.1%)	+1.5%
	How to survive the AI revolution	75/675 11.1% (8.8–13.7%)	48/572 8.4% (6.3–11.0%)	−2.7%
Are you involved in research projects on AI-based application development?	Yes, testing	61/675 9.0% (7.0–11.5%)	102/572 17.8% (14.8–21.2%)	**+8.8%**
	Yes, developing	74/675 11.0% (8.7–13.6%)	113/572 19.8% (16.6–23.3%)	**+8.8%**
	No, but planning to be involved	158/675 23.4% (20.3–26.8%)	118/572 20.6% (17.4–24.2%)	−2.8%
	No	390/675 57.8% (54.0–61.5%)	239/572 41.8% (37.7–45.9%)	−**16%**

Data are given as absolute ratio and percentage (95% CI). Differences are reported in bold characters when 95% CI are not overlapped

## Discussion

This survey highlights promises and challenges regarding the integration of AI in radiology. The rate of responders (2.0%), slightly lower than what we had in 2018 (2.8%), may be attributable not only to the saturation of surveys, but also to the self-perception of lacking sufficient knowledge about AI. Radiographers were now included and non-radiologist/non-radiology residents reached 23.6%, compared to 1.3% in 2018. As of 2018, about 50% of the respondents work at universities/teaching hospitals. Similarly, the large majority of responders (over 90%) are based in Europe.

As of 2018, breast imaging is the subspecialty thought to be most impacted by AI, followed by oncologic imaging. Conversely, general, gastrointestinal/abdominal, musculoskeletal and interventional radiology are considered to be less AI-impacted (see Fig. 3). CT is mostly predicted to be impacted by AI, followed by mammography, in agreement with the perception about breast and oncologic imaging. In fact, screening detection is the first selected application, followed by image postprocessing, protocol optimization, and oncology staging/restaging; only 5% of responders selected “prognosis” as a relevant AI application, probably due to a minor propensity to prognostication.

The perceived AI impact on job opportunities (20% increase, 28% reduction, and 52% no change), when compared to the 2018 survey (32%, 23%, and 45%, respectively) shows no overlapped percentages only for the expectations of job opportunities increase, i.e. a less optimistic vision. The subdivision into AI users (48%) and AI nonusers (52%) shows the responders’ interest in AI; 145/298 nonusers (48%) declared to be planning to use AI. About 50% of both groups do not expect AI-induced changes in job positions, with a slightly more optimistic evaluation by AI users (see Table 2), with borderline significance (*p* = 0.071). A similar trend can be observed for the association between expected variations in job positions due to AI with being/not being AI researchers (see Table 3). These results suggest that direct experience in AI usage/research may reduce the fear of job position reduction. In 2018, the primary concerns were regarding the AI model's reliability and the potential for job displacement. Now, the focus has expanded to include legal and ethical implications (29%) and data privacy vulnerabilities (20%).

The future radiologists’ AI-driven profile is perceived as more clinical (45%) or more technical (36%); more subspecialized (40%) or more open to report examinations outside their own subspecialty (36%). A similarly balanced trend characterises the perception of the AI's impact on radiological workload. This reflects the unclear consequences of AI in radiology in the mid-long term.

Importantly, 45% of responders call for the radiologist’s responsibility for AI outputs influencing clinical decision-making, while 37% foresee a “shared” responsibility. This data is mirrored by the 45% of responders who believe that patients will not accept a report made by AI alone, even though 36% think that is now too early for this prediction, without substantial change in comparison to 2018. This remains a hot topic, considering the need to keep “machines in the loop”, and avoid machines taking “humans in the loop” [3, 19]. It should be noted that the recently approved EU AI Act (https://artificialintelligenceact.eu/) mandates human oversight for all high-risk AI applications, which include any medical device.

The radiological team hopes that the time saved using AI will be dedicated to a more interactive relationship with other clinicians (40%) and patients (36%). However, to realize this hope a strong effort is needed to counteract a strategy purely aiming to maximize the number of examinations performed/reported [3].

The role of the radiological team in the development and validation of AI tools is perceived by 98% of responders (100% in 2018); “supervision of all stages” and “developing AI-based applications” were stable (64% vs 62 and 28% vs 23%, respectively), while “helping in task definition” decreased from 53% to 45%. We here should consider the declared research involvement in AI systems development and AI testing that in five years increased from 11% to 20% and from 9% to 18%, respectively. Regarding the use of AI tools in clinical practice, 48% of responders declare they are current users, mainly for CT, radiography, MRI, or mammography (only 20% in 2018), and 25% are planning to become users (30% in 2018). An increase in AI research and/or clinical use in the last five years was associated with always relevant roles of the radiological team. A question remains about cross-fertilization between data/computer scientists and the radiological world, up to complete integration of these potential “new colleagues” in the radiological team [25].

Responders demand education in AI. They confirm the topics of advantages/limitations (72% vs 69%), clinical use (62% vs 58%), “how to survive the AI revolution” (8% vs 11%), and “how to avoid the use of AI applications” (2% vs 1%). The choice of technical methods (25% vs 18%) and “how to get into the driver’s seat in using AI” (23% vs 34%) changed, showing the need to learn how AI works when already using it.

About three-quarters of responders are previous/current AI users. Of them, 53% are satisfied/very satisfied with the tools, 35% are “neutral”, and 12% are dissatisfied/very dissatisfied, showing a good-to-moderate evaluation of AI tools implemented in practice. AI companies should investigate these opinions to get feedback for improvement.

We highlight that about 80% of responders are not familiar with medical device regulation and postmarket surveillance requirements for AI tools, topics that need education efforts by radiological societies and academic institutions [12, 26, 27]. Although there is a significant difference between AI users and nonusers regarding familiarity with these regulations—approximately 30% of users and 10% of non-users are familiar (*p* < 0.001)—it is noteworthy that over 70% of AI users are not familiar, highlighting the strong demand for education in this area.

The lack of a budget dedicated to AI is noted by a majority of responders (only 9% of responders are aware of a dedicated budget). Indeed, the available evidence about the added value of AI is limited. High-quality research with well-designed clinical studies is needed, including randomized controlled trials. Both the previous and the current surveys identified similar barriers to AI implementation, such as costs, lack of validation, and integration challenges, confirming what was reported by the ESR International Forum on AI at the ECR 2021 [28].

The COVID-19 pandemic could have accelerated the adoption of AI in radiology, as supposed by nearly 60% of respondents, even though no definitive evidence exists for this.

Finally, an interest in LLMs emerges, with about 25% of the responders declaring to use LLMs often or occasionally, mainly for scientific purposes. The rate of LLMs use for clinical purposes (about 11–13%) raises concerns considering model reliability and bias, ethical implications and privacy vulnerability. It must be understood that foundation models and LLMs are not present in any current product certified for medical use. The EU AI Act does allow the use of general AI systems for high-risk applications, but actual implementations have not yet become available to test the regulatory framework’s efficacy. Therefore, any current use of generative AI or LLMs in clinical practice is “unauthorized”, with liability falling entirely on the user.

Recent regulations in the EU and the United States address the integration of AI in healthcare. The EU’s AI Act [29] mandates rigorous evaluations for high-risk AI systems, particularly in medical devices, to ensure safety, transparency, and accountability. It also requires high-quality data processing in compliance with GDPR [30]. In the United States, the 2023 Executive Order sets standards for AI, focusing on data protection, non-discrimination, and ethical use, particularly in healthcare [31]. Radiological staff members must ensure AI applications are safe and ethical, maintaining the human element in patient care. Both regions aim to balance innovation with stringent regulatory frameworks to enhance patient outcomes [32].

This survey has limitations. The low response rate and the probable self-selection imply that the herein presented data probably do not reflect the AI perceptions of the entire ESR community. Indeed, these data offer a view of the opinions and perceptions of those ESR members who currently have an interest in the impact of AI in radiology sufficient for deciding to participate in an online survey announced to require 10 min to be completed. Secondly, the survey’s voluntary nature means that individuals with strong views on AI, whether positive or negative, may be more likely to respond, potentially leading to an overrepresentation of these perspectives. Finally, the survey was administered online, which may exclude individuals who are less comfortable with digital technologies, thereby introducing a “digital” bias, even though this should not be so relevant for members of the radiological staff. Despite these limitations, the data collected provide valuable insights into the attitudes of ESR members who have a current interest in the impact of AI on radiology. Indirectly, we could hypothesize that there is no relevant fear of AI spreading within the ESR community. If such concerns existed, the opportunity to express worries and anxieties through an online survey would likely have been utilized.

Future research should focus on longitudinal studies to track AI’s evolving impact, methods to mitigate bias in AI algorithms, and exploring AI’s role in clinical decision-making. Importantly, starting from the viewpoint that AI tools are “medical devices”, the radiology community should try to evaluate their impact through randomized controlled trials. This top-level-of-evidence approach can be used for comparing the diagnostic/clinical outcome of patients randomized to AI-supported diagnoses with that of patients randomized to the traditional pathway. Additionally, ethical and legal implications such as data privacy and liability should be thoroughly investigated. For practical steps, radiology staff should engage in continuous education and training on AI advancements, adhere to ethical guidelines, collaborate with AI developers, communicate transparently with patients about AI usage, and ensure regulatory compliance. These efforts will help integrate AI effectively and ethically into radiological practice.

In conclusion, among the ESR members who participated in the survey, there is a substantial familiarity with AI technologies, as well as recognition of their potential benefits and challenges. Compared to the 2018 survey, there is no relevant change in terms of perception of AI's impact on job position opportunities, with a trend for a more optimistic evaluation from AI users/researchers. The vision of radiologists’ responsibility for AI outputs influencing clinical decision-making also remained unchanged. The use of LLMs is declared not only limited to research, highlighting the need for education in AI and its regulations.

## Supplementary information

ELECTRONIC SUPPLEMENTARY MATERIAL

## Data Availability

Data supporting the results reported in the article are available upon reasonable request to the corresponding author.

## Abbreviations

AI: Artificial intelligence
CI: Confidence intervals
EuroAIM: European Network for the Assessment of Imaging in Medicine
EuSoMII: European Society of Medical Imaging Informatics
ESR: European Society of Radiology
LLMs: Large language models

## Footnotes

1 Compared to the same question asked above, one more responder answered: “Never used”.
