# Supplementary material for: Impact of AI on radiology: a EuroAIM/EuSoMII 2024 survey among members of the European Society of Radiology
Source: Insights Imaging. 2024 Oct 7;15:240. doi: 10.1186/s13244-024-01801-w (PMC11458846; doi:10.1186/s13244-024-01801-w)
Supplement: Supplementary file 1 — ELECTRONIC SUPPLEMENTARY MATERIAL [file 13244_2024_1801_MOESM1_ESM.pdf]

# Impact of AI on radiology: a EuroAIM/EuSoMII 2024 survey among members of the European Society of Radiology

## ELECTRONIC SUPPLEMENTARY MATERIAL

Supplementary Material: full questionnaire

| Question number | Topic | Question | Answer choices |
|---|---|---|---|
| I | Status | What is your professional status? | Radiologist, Radiographer, Radiology resident, Engineer/Computer scientist, Physicist, Medical student, Other |
| II | Working place | Where do you work? | University/Teaching hospital, Hospital, Private practice, Private research center, Private company, Other |
| III | Gender | What is your gender? | Male, Female, Prefer not to say |
| IV | Age range | What is your age range? | 18–29 years, 30–39 years, 40–49 years, 50–59 years, 60–69 years, ≥ 70 years |
| V | Home country | What is your home country? | List of countries |
| VI | Subspecialty | What is your radiology subspecialty? (Up to 5 answers) | Breast, Cardiovascular, Emergency, Gastrointestinal/Abdominal, General, Head & Neck, Interventional, Molecular imaging/Nuclear, Musculoskeletal, Neuroradiology, Oncologic imaging, Paediatric, Thoracic, Urogenital |
| VII | Practiced techniques | Which radiology techniques do you frequently practice? (Up to 5 answers) | Radiography, Mammography, Ultrasound, Angiography/Fluoroscopy, CT, MRI, PET/Nuclear, Hybrid imaging, DXA, Experimental imaging (phantom/animal models), Optical imaging, Other |
| 1 | AI impact on subspecialties | Which radiological subspecialties do you foresee will be most influenced by AI in the next 5–10 years? (Select up to 3) | Breast, Cardiovascular, Emergency, Gastrointestinal/Abdominal, General, Head & Neck, Interventional, Molecular imaging/Nuclear, Musculoskeletal, |

| | | | |
|---|---|---|---|
| | | | Neuroradiology, Oncologic imaging, Paediatric, Thoracic, Urogenital |
| 2 | AI impact on techniques | Which techniques do you foresee will be the most important fields of AI applications in the next 5–10 years? (Select up to 3) | Radiography, Mammography, Ultrasound, Angiography/Fluoroscopy, CT, MRI, PET/Nuclear, Hybrid imaging, DXA, Experimental imaging (animal models), Optical imaging, Other |
| 3 | AI applications | Which of the following AI applications do you think are most relevant as aids to the radiological profession? (Select up to 3) | Imaging protocol optimisation, Image post-processing, Detection in asymptomatic subjects (screening), Detection of incidental findings, Lesion characterization/diagnosis in symptomatic subjects, Staging/restaging in oncology, Support for structured reporting, Quantitative measure of imaging biomarkers, Prognosis, Other |
| 4 | Job opportunities | Do you foresee an AI impact on professional radiologist’s life in terms of the number of job positions in the next 5–10 years? | No, Yes, job positions will be reduced, Yes, job positions will increase |
| 5 | Radiologist’s duties | In the next 5–10 years, will the use of AI-based applications make radiologists' duties more technical, more clinical, or unchanged? | More technical, More clinical, Unchanged, Other |
| 6 | Subspecialty focus | Do you think that, in the next 5–10 years, the use of AI-based applications will help radiologists report examinations outside their field of subspecialization? | No, radiologists will be more focused on radiology subspecialties, Yes, radiologists will be less focused on radiology subspecialties, The rate of dedication to subspecialties will remain unchanged |
| 7 | Workload | Do you foresee an AI impact on professional radiologist’s life in terms of total reporting workload in the next 5–10 years? | No, Yes, it will increase, Yes, it will be reduced |

| | | | |
|---|---|---|---|
| 8 | Legal responsibility | In the next 5–10 years, who will take the legal responsibility for AI-system output? | Radiologists, Other physicians (e.g., clinicians requesting the imaging study), Developers of AI applications, Insurance companies, Shared responsibility, Other |
| 9 | Patient acceptance | In the next 5–10 years, will patients mostly accept a report from AI applications without supervision and approval by a physician? | Yes, No, Difficult to estimate at present |
| 10 | Radiologist-patient relationship | How will the relationship between the radiologist and the patient change because of AI introduction? | More impersonal, More interactive, Unchanged |
| 11 | Role in AI development | What will be the role of radiologists in developing/validating AI applications for medical imaging? | None, Provide labelled images, Help in task definition, Develop AI-based applications, Supervise all stages needed to develop an AI-based application |
| 12 | AI education | Should radiologists be educated on: | Technical methods (e.g., machine/deep learning algorithms), Advantages and limitations of AI applications, Clinical use of AI applications, How to get into the driver's seat in using AI, How to avoid the use of AI applications, How to survive the AI revolution |
| 13 | Use of saved time | If AI will allow saving working/reporting time, should radiologists use the saved time for interacting with: | AI developers (e.g., engineers, computer scientists), Other radiologists, Other clinicians, Patients, Administrators |
| 14 | Current use of AI | Are you utilizing AI-based products or services in your clinical practice? | Yes, No, but planning to utilise, No |
| 15 | AI research involvement | Are you involved in research projects on AI-based application development? | Yes, testing, Yes, developing, No, but planning to be involved, No |

| | | | |
|---|---|---|---|
| 16 | AI satisfaction | How satisfied are you with the performance and impact of certified AI tools in your clinical practice? | Never used, Very satisfied, Satisfied, Neutral, Dissatisfied, Very dissatisfied |
| 17 | AI utilisation | Please specify on which radiological modality/ies the certified AI tools you are using are applied | Never used, Radiography, Mammography, Ultrasound, Angiography/Fluoroscopy, CT, MRI, PET/Nuclear, Hybrid imaging, DXA, Others |
| 18 | Medical Devices Regulation knowledge | Are you familiar with the classification under the Medical Devices Regulation and the post-market surveillance requirements for the certified AI tool you are using? | Never used, Yes, No, I do not know the classification under the Medical Devices Regulation, I do not know the post-market surveillance requirements |
| 19 | AI knowledge | How do you keep yourself informed about AI? | Books, Scientific papers, Conferences/Congresses, Newsletters, Social media, Colleagues, AI itself, Other |
| 20 | AI budget | Does your department have a dedicated budget for AI? | Yes, No, I do not know |
| 21 | AI research contribution | What was your contribution to AI research in medical imaging? | Proponent (identifier of clinical needs), Developer (direct interaction and collaboration with data scientists), Data selector and provider<br>Image annotation/segmentation (and or provider ground truth/reference standard), Supervision of training and internal validation (reviewing and testing AI models), Collaboration in external validation, Cooperation in writing of scientific/clinical reports, Not involved in any AI research. |
| 22 | Covid-19 pandemic impact | Do you believe that the Covid-19 pandemic accelerated the development or implementation of AI | Yes, No, I do not know |

| | | | |
|---|---|---|---|
| | | research in medical imaging? | |
| 23 | AI implementation barriers | What do you think might be the main potential barriers for AI implementation in clinical practice? | Costs/lack of budget, Information technology and systems integration, Lack of validation/scientific evidence, Lack of vision/policy/ownership, Legal issues, Others |
| 24 | LLMs clinical practice | Do you use Large Languages Models (e.g. ChatGPT) in your clinical practice? | Yes, often, Yes, occasionally, No, but I am interested, No, and I am not interested |
| 25 | LLMs clinical practice | For what settings do you mainly use Large Languages Models (e.g. ChatGPT) in clinical practice? (Up to 3 answers) | Risk assessment, Screening/Diagnosis, Treatment planning and management, Image annotation and reporting, Research activities/scientific writing, Literature review, Never used |
| 26 | LLMs benefits | How do you perceive the main potential benefits of using Large Languages Models (e.g. ChatGPT) in radiology? (Up to 3 answers) | Improved diagnostic accuracy, Enhanced efficiency in image interpretation (reporting prioritising), Enhanced efficiency imaging procedure execution, Access to up-to-date medical literature and research, Cost savings. Not sure/No opinion |
| 27 | LLMs concerns | What are your main concerns or reservations about incorporating Large Language Models (e.g., ChatGPT) into clinical practice? (Up to 2 answers) | Data privacy vulnerability, Legal and ethical implications, AI model reliability and bias, Negative impact on radiological staff, No concerns/reservations, Other |
| 28 | AI generative models | Have you used AI imaging generative models (e.g., Midjourney or Adobe Firefly) to generate medical images or enhance image quality in your clinical practice or for academic purposes? | Yes, No, but I am interested, No, and I am not interested |

Supplementary File: Data on respondent country distribution

| Country | Responders number | Percentage |
|---|---|---|
| 1. Italy | 95 | 16,6% |
| 2. Greece | 47 | 8,2% |
| 3. Germany | 44 | 7,7% |
| 4. Portugal | 33 | 5,8% |
| 5. Romania | 30 | 5,2% |
| 6. United Kingdom | 24 | 4,2% |
| 7. Netherlands | 22 | 3,8% |
| 8. Spain | 22 | 3,8% |
| 9. Switzerland | 19 | 3,3% |
| 10. Austria | 16 | 2,8% |
| 11. France | 14 | 2,4% |
| 12. Poland | 13 | 2,3% |
| 13. Turkey | 13 | 2,3% |
| 14. Ukraine | 13 | 2,3% |
| 15. Sweden | 12 | 2,1% |
| 16. Belgium | 11 | 1,9% |
| 17. Bulgaria | 9 | 1,6% |
| 18. Serbia | 9 | 1,6% |
| 19. Croatia | 8 | 1,4% |
| 20. Denmark | 8 | 1,4% |
| 21. Finland | 8 | 1,4% |
| 22. Norway | 8 | 1,4% |
| 23. Georgia | 7 | 1,2% |
| 24. Moldova | 7 | 1,2% |
| 25. Cyprus | 5 | 0,9% |
| 26. Ireland | 5 | 0,9% |
| 27. Hungary | 4 | 0,7% |
| 28. India | 4 | 0,7% |
| 29. Latvia | 4 | 0,7% |
| 30. Malta | 4 | 0,7% |
| 31. Russia | 4 | 0,7% |
| 32. Slovenia | 4 | 0,7% |

| | | |
|---|---|---|
| 33. Armenia | 3 | 0,5% |
| 34. Herzegovina | 3 | 0,5% |
| 35. Iran | 3 | 0,5% |
| 36. Kazakhstan | 3 | 0,5% |
| 37. Lithuania | 3 | 0,5% |
| 38. Republic | 3 | 0,5% |
| 39. Azerbaijan | 2 | 0,3% |
| 40. Luxembourg | 2 | 0,3% |
| 41. Macedonia | 2 | 0,3% |
| 42. Pakistan | 2 | 0,3% |
| 43. Slovakia | 2 | 0,3% |
| 44. Albania | 1 | 0,2% |
| 45. Argentina | 1 | 0,2% |
| 46. Australia | 1 | 0,2% |
| 47. Belarus | 1 | 0,2% |
| 48. Canada | 1 | 0,2% |
| 49. China | 1 | 0,2% |
| 50. Egypt | 1 | 0,2% |
| 51. Estonia | 1 | 0,2% |
| 52. Ethiopia | 1 | 0,2% |
| 53. Iceland | 1 | 0,2% |
| 54. Israel | 1 | 0,2% |
| 55. Lebanon | 1 | 0,2% |
| 56. Montenegro | 1 | 0,2% |
| 57. Nigeria | 1 | 0,2% |
| 58. Republic | 1 | 0,2% |
| 59. United States of America | 1 | 0,2% |
| 60. Uzbekistan | 1 | 0,2% |
| 61. Vietnam | 1 | 0,2% |
